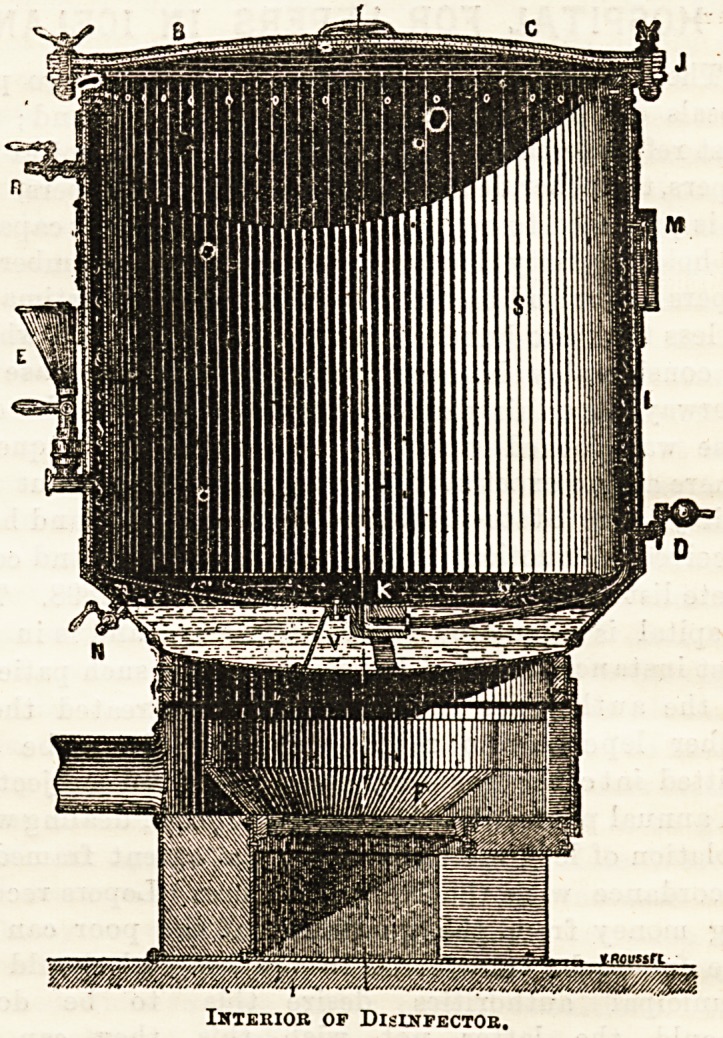# Practical Departments

**Published:** 1895-12-28

**Authors:** 


### PRACTICAL DEPARTMENTS.

### A NEW FRENCH DISINFECTING MACHINE.

A disinfecting stove has recently been invented by Messrs. Vaillard and Besson, two professors attached to the medical school in connection with the Military Hospital, Val-de-Grâce, in Paris, who claim for their invention (1) utmost simplicity of structure, (2) complete and certain efficiency, (3) almost automatic working, by which thorough disinfection is guaranteed and all risk of accident avoided. The apparatus consists of two parts; the furnace below, and above, and resting on it, the disinfecting] chamber, which is made of double plates of galvanised steel, with a small intervening space at the sides, whilst below is a considerable space which serves as a boiler, V. This boiler is filled with water through the funnel, E, and the tap, N, marks the level of the water. The inner plate can be removed when the boiler wants repairing. When the fire is lighted steam is generated in the boiler and ascends between the two plates, enters the upper part of the chamber, and passes out again below. To facilitate this circulation the cylinder, S, is pierced with small holes through which the steam passes. In the floor of the cylinder is a round opening fitted with a galvanised iron pipe, V D, for the escape of steam; but there is no communication between the boiler and the disinfecting chamber except above. The top of the apparatus is closed by a strong cover firmly screwed down by bolts, and the whole machine is covered by a jacket of felt inside, and thin steel outside. The gauge, M, shows pressure and temperature inside the chamber, and the tap, R, allows steam to escape when required. To use the disinfecting machine, pour water into the funnel, E, until it begins to run out at the tap below, E, which must then be closed. The articles to be disinfected are next put into the chamber and covered with a sheet to prevent any damping by the very small quantity of steam which condenses inside the cover. The cover is then firmly screwed down with a "key," the valve D is opened, the fire lighted, and the whole apparatus heated. When steam begins to escape by the valve D it is allowed to continue to do so for five minutes, and then the valve is closed, and the pressure and temperature are raised to the required point, viz., 112 deg. C., as shown by the gauge, and this temperature can be maintained for fifteen to twenty minutes merely by keeping up a good fire. Any disinfecting fluid can be substituted in the boiler for water. Disinfection is complete in twenty minutes, and pressure is gradually reduced by swing tap R, and the door of the furnace opened to lessen the heat. When the gauge indicates zero the disinfecting chamber is opened, and its contents at once removed.

Interior of Disinfector.

The Vaillard and Besson Disinfector.

## Figures and Tables

**Figure f1:**
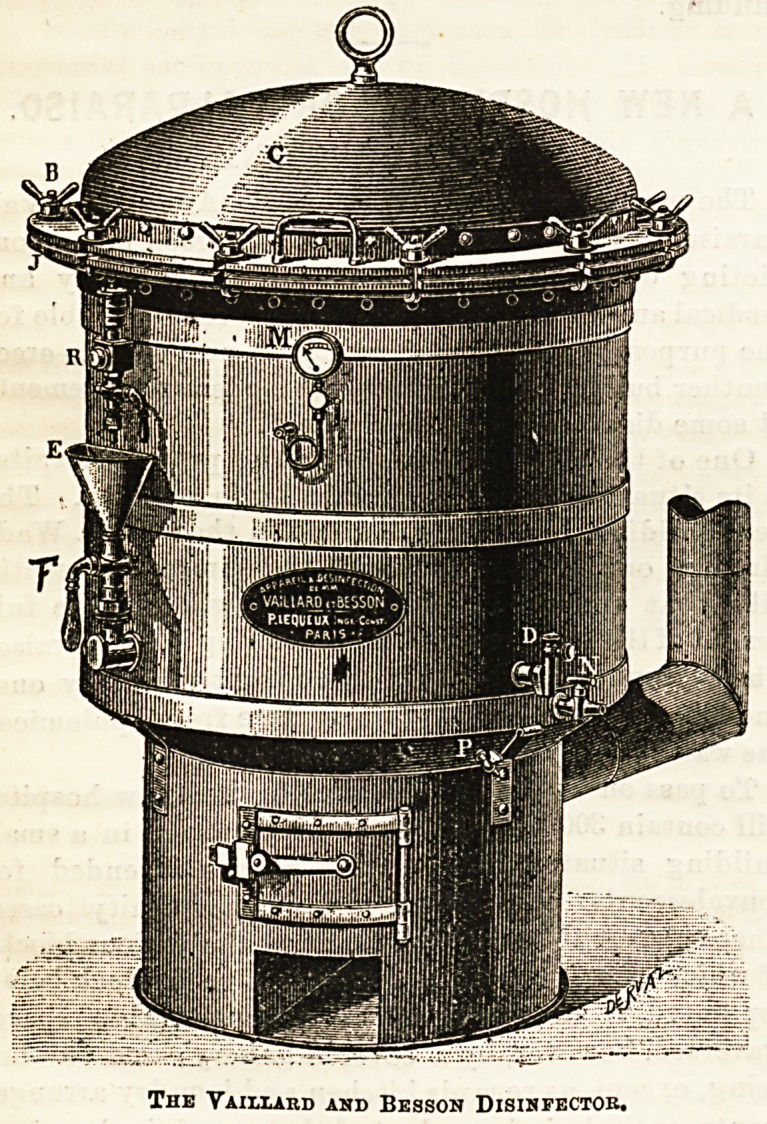


**Figure f2:**